# Determination of reference values for tear production and intraocular pressure in Pygoscelis penguins of the Antarctic Peninsula

**DOI:** 10.1186/s12917-023-03794-y

**Published:** 2023-11-10

**Authors:** Latife Cakir Bayram, Cafer Tayer Isler, Görkem Ekebas

**Affiliations:** 1 Department of Pathology, Faculty of Veterinary Medicine, Erciyes University, Kayseri, 38280 Turkey; 2 Department of Surgery, Faculty of Veterinary Medicine, Hatay Mustafa Kemal University, Hatay, Turkey

**Keywords:** Intraocular pressure, *Pygoscelis* penguins, Rebound tonometry, Schirmer's tear test, Tear production, Tonovet ®

## Abstract

**Background:**

According to the literature review, this is the first study investigating tear production (TP) and intraocular pressure (IOP) in the *Pygoscelis* penguins living in their natural habitat. The study aimed to establish normal values for standard ocular tests in the genus *Pygoscelis*, namely, the Adélie (*Pygoscelis adeliae*), gentoo (*Pygoscelis papua*), and chinstrap (*Pygoscelis antarctica*) penguins, in four different islands of Antarctica. Sampling was made by specifically using the left eye of the penguins. The Schirmer's tear test type I (STT-I) and the Tonovet® (rebound tonometer) were used to measure the TP and the IOP, respectively.

**Results:**

The mean TP and IOP values of 129 Adélie, chinstrap, gentoo, and 120 adult Adélie, gentoo penguins were determined as 10.2 ± 4.0 mm/min and 38.9 ± 13.2 mmHg, respectively. No statistical difference was detected between the penguin species for the mean IOP values, while the difference was determined in all the locations. However, statistical differences in the mean TP values were determined between all locations.

**Conclusion:**

The results of this study provide a reference range of Schirmer's tear test (STT) and IOP values in *Pygoscelis* penguins and show that the IOP is significantly affected by locations. This result can be attributed to the harsh climatic conditions of the Antarctic Peninsula that change very quickly. The described data may help diagnose clinical pathological findings in Pygoscelis penguins.

The STT and rebound tonometry appears to be safe and reproducible methods in *Pygoscelis* penguins, as the results were obtained quickly and were well tolerated by the birds.

Based on our results, we propose that similar studies can be initiated in crowded colonies of three penguin species of this genus on the Antarctic Peninsula, the southern Shetland Islands, and other frequently visited islands in Antarctica.

## Highlights

• This study is the first to investigate tear production (TP) and intraocular pressure (IOP) in *Pygoscelis* penguins.

• TP value was obtained by Schirmer's tear test type I (STT- I), while the IOP was measured with the help of Tonovet® (rebound tonometer).

• No clinical macroscopic findings affecting the eyelids, third eyelid, cornea, or ocular eye surface were found during the clinical examination of the penguins.

• There was no difference in TP values between species of this genus and locations. There was no statistically significant difference between species in IOP mean values. However, a significant difference was noticed among the locations.

• This study indicated that IOP in Pygoscelis penguins was significantly affected by location.

## Introduction

Ocular health bears significance for survival and self-sustainment. Therefore, regular ocular examination not only enables the collection of essential medical data and the protection of the health of animals under professional care but also constitutes an integral part of monitoring wild animals [1–3]. *Pygoscelis* penguins are found mainly in the higher latitudes of the sub-Antarctic and the Antarctic Peninsula. According to the International Union for Conservation of Nature (IUCN), the general threat status of all three *Pygoscelis* species is of minor concern, while according to Dunn et al., the Adélie (*Pygoscelis adeliae*), and chinstrap (*Pygoscelis antarctica*) penguins are declining. Still, gentoo penguins (*Pygoscelis papua*) are increasing regionally [4]. Eyesight is critical for penguins as it aids in migration, orientation, and foraging [5]. Thus, vision disorders adversely affect the capability of these animals to adapt to their physical and social environments. In penguins, interpreting ocular findings and performing diagnostic tests are exceedingly tricky [6]. Ocular disorders are relatively common, especially among free-living birds [7]. While numerous bacteria, viruses, fungi, and parasites have been isolated from the avian ocular surface in various diseases [8], only a few studies are available on the ophthalmic parameters of several penguin species, including ocular bacterial flora, IOP, and STT [5, 6, 9–11]. In a study by Swinger et al. (2009), the ocular bacterial biota and ophthalmic parameters of 28 captive penguins kept at a zoo were investigated [12]. However, there is no published study of three species belonging to the *Pygoscelis* genus or on the ocular infections of penguins. The first documented report is of the unilateral pyogranulomatous ocular lesion in a gentoo penguin chick living in its natural habitat in Antarctica [13].

STT and IOP measurements are the main diagnostic tools for multiple ocular diseases. There is a need to establish species-specific reference values for the STT and IOP [14–16], considering differences between avian species. The amount of TP serves as an essential parameter for the assessment of the pathological condition of the ocular surface. The STT has been the gold standard for determining the amount of TP [10, 12, 17]. Furthermore, the STT is the most used test for diagnosing ocular diseases in veterinary medicine [18].

IOP can be described as the balance between the production and secretion of aqueous humor. Abnormally high or low IOP indicates ocular diseases, such as uveitis and glaucoma [10, 12]. Contact tonometry is repeatable and has provided almost precise results over the past decade. Nonetheless, the use of TonoVet® (Icare Finland, Oy) (McLennan), ICare® Tonovet (TV01; Icare Finland Oy, Helsinki, Finland) (Gloe Shawna), and TonoVet Plus® (Icare) rebound tonometers for the measurement of IOP in domestic, laboratory, exotic and wild animals has gained popularity.

Rebound tonometry requires only an instant contact between the probe and the corneal surface [19–21]. As a minimally invasive technique, rebound tonometry can be safely performed for corneal diameters as small as 1.4 mm [11, 22, 23]. Rebound tonometry does not require topical anesthesia and measures the deceleration of the probe, which is rapidly and repeatedly bounced against the cornea [24–26]. The Tonovet for veterinary usage has been specifically designed for animal use. It generates calibration curves for IOP measurement in small animals using different settings (D for dogs, H for horses, and P for other species) [27].

Normal IOP and STT values have been determined with diagnostic ophthalmic tests in species of the order Sphenisciformes, including the macaroni penguin (*Eudyptes chrysolophus*) [6, 23], southern rockhopper penguin (*Eudyptes chrysocome*) [6], black-footed penguin (*Spheniscus demersus*) [3, 24], Humboldt penguin (*Spheniscus humboldti) *[12, 16], gentoo penguin (*Pygoscelis papua*), king penguin (*Aptenodytes patagonicus*), and chinstrap penguin (*Pygoscelis antarctica*). Typical STT values [28–30] and IOP values have been reported for various domestic and wild animals [31–33]. However, minimal information is available on these ocular parameters in penguins living in their natural Antarctic habitat.

Statistically, significant differences have been reported between IOP values measured with the Tonovet® rebound tonometer concerning animal species, age, and ocular pathologies [16, 22, 34]. Some studies have reported the absence of statistically significant gender-related differences between STT values and Tonovet® rebound tonometer-produced IOP values in young and old birds [35, 36]. Some other studies have suggested that STT and IOP values in animals do not significantly differ for age, gender, or the left/right eye [30, 34, 35, 37].

Normal IOP and STT values for the Humboldt penguin [12, 16], macaroni penguin [6, 34], southern rockhopper penguin [6], black-footed penguin, gentoo penguin, king penguin, and chinstrap penguin [3, 24, 32] have been reported. Despite the availability of literature reports on STT and IOP in various penguin species, most of the populations investigated in these reports cover captive penguins kept under professional care at zoos or wildlife rehabilitation centers [24, 34, 38]. On the other hand, there is a lack of data on normal STT-I and Tonovet® rebound tonometer-produced (Icare® Oy, Finland (TV01)) IOP values in *Pygoscelis* penguins.

This study aimed to establish typical values for standard ocular tests in the genus *Pygoscelis,* namely, the Adélie, gentoo and chinstrap (penguins, in four different islands of Antarctica. Sampling was made specifically from the left eye of the penguins. STT-I and the Tonovet® (rebound tonometer) were used to measure the lacrimal production and intraocular pressure, respectively.

Therefore, this study aimed to establish typical values for standard ocular tests in the genus *Pygoscelis,* namely, the Adélie, gentoo and chinstrap penguins, in four different islands of Antarctica, and to establish reference ranges of TP and IOP values for Adélie penguin, chinstrap penguin and gentoo penguin The mean STT and IOP values determined for each *Pygoscelis* penguin species in this study will serve as reference values for captive penguins in zoos and aquariums and future studies on the Antarctic Peninsula.

## Results

The mean tear production amounts of the gentoo, Adélie, and chinstrap penguin species were calculated as 9.8 ± 3.9 mm/min, 11.1 ± 2.3 mm/min, and 11.9 ± 5.0 mm/min, respectively.

For the mean values of STT, the *p*-value was found to be 0.091 in the ANOVA test performed for three species, and the difference was calculated to be insignificant (*p* > 0.05) (Table 1). When the Independent sample t-test was performed, excluding the Adélie penguin (*n* = 7), the *p*-value was calculated as 0.037 for between the gentoo (*n* = 104) and chinstrap (*n* = 18) species (*p* < 0.05).

**Table 1:** Comparison of TP amounts (mm/min) by species

Species	n	STT-1		95% CI		p
		**Mean**	**SD**	**Lower**	**Upper**	
Adélie	7	11.1	2.3	9.0	13.3	0.091
Chinstrap	18	11.9	5.0	9.5	14.4	
Gentoo	104	9.8	3.9	9.0	10.6	
Total	129	10.2	4.1	9.5	10.9	

*SD* Standard deviation of the mean, *95% CI *95% confidence interval for mean STT-1 value

*p * > 0.05

The mean IOP values for the Gentoo and Adélie species examined in this study were 39.3 mmHg ± 11.6 and 38.4 mmHg ± 14.8, respectively (*p* > 0.05).

There was no statistically significant difference between species in terms of mean IOP values (Table 2).

**Table 2:** Comparison of IOP values (mmHg)by species

Species	n	IOP		95% CI for Mean		p
		**Mean**	**SD**	**Lower**	**Upper**	
Adélie	7	38.4	14.8	24.7	52.2	0.854
Gentoo	113	39.3	11.6	37.1	41.4	
Total	120	38.9	13.2	37.0	41.2	

*SD* Standard deviation of the mean, *95% CI *95% confidence interval for mean IOP value

*p * > 0.05

The mean STT value calculated for measurements made in six different regions in 129 adult penguins was 10.2 ± 4.1 mm/min in the 3–25 mm/min range. At the Harmony point, the highest value of STT was measured at 25 mm/min. This value varies between 15–18 mm/min in other locations. Mean STT values by location were insignificant (Table 3).

**Table 3:** Comparison of TP amounts (mm/min) by locations

Locations	n	STT-1		95% CI		p
		**Mean**	**SD**	**Lower**	**Upper**	
O'Higgins	17	9.4	3.9	7.4	11.4	0.088
Harmony	17	12.2	5.0	9.6	14.8	
Ardley I	29	9.4	3.5	8.1	10.7	
Ardley II	17	11.2	3.8	9.2	13.2	
Ardley III	24	9.5	3.6	8.0	11.0	
Lions Rump	25	10.1	4.4	8.3	11.9	
Total	129	10.2	4.1	9.5	10.9	

*SD* Standard deviation of the mean, *95% CI *95% confidence interval for mean STT-1 value

*p*  > 0.05

 Descriptive values of IOP by locations and penguin species are presented in Tables 4 and 5. The mean IOP of 120 penguins was 38.9 ± 13.2 mm Hg. The highest value was measured in the Ardley III as 69 mm Hg. This value varies between 50–63 mm Hg in other locations. Due to adverse weather conditions, only one value could be measured in the chinstrap penguin. For the same reason, IOP values could not be calculated at Harmony Point.

**Table 4:** Comparison of IOP values (mmHg) by locations

Locations	n	IOP		95% CI for Mean		p
		**Mean**	**SD**	**Lower**	**Upper**	
O'Higgins	17	35.4^b^	10.3	30.1	40.7	0.001
Ardley I	29	37.3^b^	8.0	34.3	40.3	
Ardley II	17	36.4^b^	9.6	31.5	41.3	
Ardley III	33	51.1^c^	8.2	48.2	54.0	
Lions Rump	25	29.9^a^	9.1	26.2	33.7	
Total	120	38.9	13.2	37.0	41.2	

*SD *Standard deviation of the mean, *95% CI *95% confidence interval for mean IOP value

^a, b, c^: Means with a different superscript are significantly different at an alpha level of 0.05 according to Tukey’s HSD test

*p* < 0.001

**Table 5:** Geographical position (Latitude and longitude of study locations) with ophthalmic examination types and the number of penguins included in the ophthalmic measurement

**Study Locations**	**Number of penguins**^**†**^	**Latitude S** **DD**°**MM**' **SS**"	**Longitude W** **DD**°**MM**' **SS**"
1^*^	^ab^17	63° 19′ 20"	57° 54′ 04"
2	^ab^25	62° 07′ 57"	58° 08′ 09"
3	^b^ 17	62° 18′ 31"	59° 12′ 34"
4	^ab^29	62° 18 ′34”	58° 55′ 34”
5	^ab^17	62° 12′ 35”	55° 55′ 43”
6	^a^33,^b^24	62° 12′ 34”	58° 56′ 01”

*DD MM SS* Degree Minute Second

^*^: Study Locations Numbers:1. Cabo Legoupil/ General Bernardo O’Higgins Base. 2. Lion Rump area, King George Island ASPA (151). 3. Harmony point /Nelson Island (ASPA 133).4–6. Ardley island (ASPA 150). 4.Ardley I. 5. Ardley II.6. Ardley III

^†^: Type of Ophthalmic examination: ^a^Intraocular pressure measurement with Icare® rebound tonometer

^b^Measurement of tear secretion by Schirmer’s Tear Test -1

There was a significant difference between Ardley III-Bernardo O'Higgins Base (*p* < 0.001), Ardley III-Ardley I (*p* < 0.001), Lions Rump-Ardley I (*p* = 0.023 < 0.05), Ardley III-Ardley II (*p* < 0.001) and Lions Rump-Ardley III (*p* < 0.001).

## Discussion

This is the first study reporting IOP and STT in free-ranging *Pygoscelis* penguins. In the present study, while there was no significant difference in STT values between species and sampling locations, it is noteworthy that there was a substantial difference of *p *< 0.001 between IOP value and locations**.**

Based on the literature review, there is no previous study on ophthalmic findings of penguins living in their natural habitat; therefore, diagnostic ophthalmic reference values have not been established for these animals. As all the studies conducted to date have been performed under professional care in zoos or wildlife rehabilitation centers in controlled environments, [16] there is a need for further research.

It has been reported that developing a standardized tear test for birds may not be possible due to anatomical and physiological differences observed between species for tear drainage and lacrimal ducts [39]. Since few ophthalmologic studies have been previously conducted in penguins due to the difficulty of applying diagnostic tests in these birds, there is a lack of information on ocular examination and data interpretation for penguins [40–42]. Establishing reference ranges for each species is essential in avoiding erroneous diagnostic interpretations during ophthalmic examination [43–45].

Studies on IOP and STT values in penguin species belonging to the order Spheniscus have been conducted in animals kept under professional care in artificial marine and freshwater environments at either wildlife rehabilitation centers or zoos (Table 6) [12, 23, 32]. In the study on macaroni penguins and southern rockhopper penguins kept at zoos and aquariums in North America, Woodhouse et al. [23] assessed the impact of multiple factors on IOP values in penguins, including husbandry conditions, the presence/absence of cataracts and concurrent ocular pathologies, and the body position during physical restraint. To our knowledge, this is the first study on STT and IOP in *Pygoscelis* penguins, the Adélie, chinstrap, and gentoo, living in their natural habitat on the southern Antarctic islands. In contrast with the previous controlled studies conducted in a closed environment, this study was conducted in the open air, the natural marine habitat of penguins. Measurements were performed on the animals during their daily routine while exposed to dust, ocean water spray, intense winds, snow, and abrupt weather changes at an average environmental temperature of -4 ^0^C. When assessing the impact of species-specific anatomical and physiological differences, stress, and geographical conditions on parameters such as STT in raptorial birds, it should be noted that data comparisons can be made only under optimal conditions [31]. Considering the differences in IOP values between species (Table 7), in agreement with previous studies, the higher IOP values found in *Pygoscelis* penguins were attributed to the fact that these species dive up to 30 m below the ocean surface. Thus, their corneas are exposed to elevated levels of external pressure, and the higher IOP values have been implicated as an adaptive function associated with underwater foraging. The only study previously conducted on these ocular parameters in populations living in their natural habitat was conducted in the Punta San Juan Conservation Area in Peru [16]. This study presents, for the first time, IOP values detected in *Pygoscelis* penguins. It was observed that IOP values varied with the age of the penguin, as well as with the location. Previous studies by Swinger and Mercado [3, 12] determined higher IOP values that fell within a more extensive range than those previously detected in zoo animals. Suggesting that the higher IOP values they had detected were an adaptation of the animals to the higher atmospheric pressure they were exposed to during underwater dives, these researchers also indicated the necessity for further research to confirm their hypothesis [3]. In the present study, while no statistically significant difference was determined between the penguin species for the mean IOP values (*p* = 0.854) (Table 2), the study locations significantly differed for both the STT-I and IOP values (*p* < 0.05, *p* < 0.001) (Tables 3 and 4).

**Table 6:** Reference values of intraocular pressure (IOP) and tear production (TP) from the healthy eyes of 8 breeds of captivity and non-Antarctic penguins

	**TP (mm)/min**				**IOP, mm Hg**				**References**
**Species**	**Technique**	**mean**	** ± **	**SD, range**	**Tonometer**	**mean**	** ± **	**SD, range**	
Humboldt penguin *(Spheniscus humboldti)*	(STT-I)	9	±	4, (2–20)	(TP-D)	28	±	9, (3–49)	Sheldon, et al. (2016) [16]
	(STT-I)	6.45	±	2.9, (1–12)	(TV-XL)	20.4	±	4.1	Swinger RL, 2009 [12]
Macaroni penguin *(Eudyptes chrysolophus)*	(STT-II)	12.1	±	5.43	(TV-D)	29.1	±	7.1	Bliss CD, 2015 [6]
			±		(TV-D)	42.0	±	9.7	Woodhouse SJ. 2016 [23]
Rockhopper penguin *(Eudyptes chrysocome)*	(STT-II)	11.0	±	3.96	(TV-D)	24.1	±	5.09	Bliss CD, 2015 [6]
					(TV-D)	32.9	±	6.2	Woodhouse SJ. 2016 [23]
Black-footed penguin *(Spheniscus demersus)*					TV-D; Icare®)	31.8	±	3.3	Gonzalez-Alonso‐Alegre EM 2015 [24]
					TV-D	30.4	±	4.3 OD	Mercado, J. A.2010 [3]
					TV-D	28.1	±	6.8 OS	
					TV-H	25.06	±	4.35 OD	
					TV-H	25.05	±	5.56 OS	
• Southern Rockhopper *(Eudyptes chrysocome)* • Gentoo penguin *(Pygoscelis papua)* • King penguin *(Aptenodytes patagonicus)* • Chinstrap penguins *(Pygoscelis antarctica)*					Tono- Pen XL®;	6	±	4–13	Church, M. 2018 [32] Hadden, PW. 2022 [46]
					TonoVet®-	16	±	4–22	

*OD* Right Eye, *OS* Left Eye

**Table 7:** Summary of TP and IOP data from studies Icare® rebound tonometry and the Schirmer’s tear test in avian species

**Species**	**TP (mm/min)**				**IOP (mmHg)**				**References**
	**mean**	** ± **	**SD**	**range**	**mean**	** ± **	**SD**	**Range (technique)**	
American flamingo (Phoenicopterus ruber)					11.1	±	2.3	8–21 (56) OS (TonoVet®-P)	Molter et al. 2014 [11]
					10.9	±	1.8	7–15 (28) OD (TonoVet®-P)	Molter et al. 2014 [11]
	12.3	±	4.5	4–20 (18)	9.5	±	1.7	7–13 (16) (TonoVet®-P)	Meekins et al. 2015 [14]
					16.1	±	4.2	(Tonopen XL)	Meekins et al. 2015 [14]
Barn owl17 (Tyto alba)					10.8	±	3.8	5–16 (6) (TonoVet®-D)	Reuter et al. 2011 [22]
Common kestrel (Falco tinnunculus)					9.8	±	2.5	4–15 (141) (TonoVet®-D)	Reuter et al. 2011 [22]
Eurasian sparrowhawk17 *(Accipiter nisus)*					15.5	±	2.5	10–23 (47) (TonoVet®-D)	Reuter et al. 2011 [22]
Long-eared owl17 *(Asio otus)*					7.8	±	3.2	4–13 (21) (TonoVet®- D)	Reuter et al. 2011 [22]
Northern goshawk17 *(Accipiter gentilis)*					18.3	±	3.8	12–29 (58) (TonoVet® -D)	Reuter et al. 2011 [22]
Peregrine falcon17 *(Falco peregrinus)*					12. 75	±	8.00	5–21 (7)	Reuter et al. 2011 [22]
Red kite17 *(Milvus milvus)*					13.0	±	5.5	4–19 (8) (TonoVet® -D)	Reuter et al. 2011 [22]
White-tailed sea eagle17 *(Haliaeetus albicilla)*					26.9	±	5.8	17–41 (29)	Reuter et al. 2011 [22]
Tawny owl *(Strix aluco)*					9.4	±	4.1	3.0–17 (27)	Reuter et al. 2011 [22]
Long-eared owl *(Asio otus)*					7.8	±	3.2	4.0–13.0 (21) (TonoVet®-D)	Reuter et al. 2011 [22]
Eurasian Sparrowhawk *(Accipiter nisus)*					15.5	±	2.5	10.0- 23.0 (47)	Reuter et al. 2011 [22]
Common Buzzard *(Buteo buteo)*					26.9	±	7.0	14.0- 44.0 (86)	Reuter et al. 2011 [22]
Eurasian eagle owl *(Bubo bubo)*					10.5	±	1.6	7–14 (20) (TonoVet ®-P)	Jeong et al. 2007 [31]
					9.35	±	1.81	(TonoPen®XL ®)	Jeong et al. 2007 [31]
Bald eagle12 *(Haliaeetus leucocephalus)*	14	±	2	8–19 (32)	21,5	±	1.7	(Tonopen® XL)	Kuhn et al. 2013 [29]
Amazon parrots *(Amazona ventralis)*	7.9	±	2.6	0–13 (48)					Storey et al. 2009 [47]
Common buzzard *(Buteo buteo)*	12.5	±	2.7					(20) (Tonopen® XL)	Barsotti et al. 2013 [48]
Eurasian tawny owl *(Strix aluco)*	3.12	±	1.92					(20)	Barsotti et al. 2013 [48]
Little owl (*Athene noctua)*	3.5	±	1.96					(20) (Tonopen® XL)	Barsotti et al. 2013 [48]
European kestrel* (Falco tinnunculus)*	6.20	±	3.67					(20)	Barsotti et al. 2013 [48]
Ostrich *(Struthio camelus)*	16.3	±	5 (40)					13.0–22.5 (40) (Tono-Pen Vet®)	Ghaffari et al. 2012 [28]
Duck	6.2	±	2.2 (96)		10.2	±	2.2		Ansari Mood et al. 2017 [10]
Geese	5.5	±	2.6 (104)		9.1	±	2.0	TonoVet ®-P, Icare	Ansari Mood et al. 2017 [10]
Pigeon					6.0	±	0.9	OD 3–9 (100)	Ansari Mood et al. 2016 [30]
					6.1	±	1.0	6.1 ± 0.9 OS TonoVet ®-P (100)	Ansari Mood et al. 2016 [30]
Cooper’s Hawk* (Accipiter cooperi)*				9.0–12.0	10.7	±	1.4	9.0–12.0 (TonoVet®-Icare-P) (6)	Labella et al. 2012 [21]
Turkey vulture* (Cathartes aura)*				10.0–12.0	11.7	±	1.0	10.0–12.0 (6)	Labella et al. 2012 [21]
Red -Taileed Hawk (*Buteo jamaicensis)*				14.0–34.0	19.8	±	4.9	14.0–34.0 (44)	Labella et al. 2012 [21]
American Kestrel* (Falco sparverius)*				8.0–9.0	6.8	±	1.7	5.0–9.0 (8)	Labella et al. 2012 [21]
Eastern Screech owl *(Megascops asio*)					6.3	±	1.3	5.0–8.0 (4)	Labella et al. 2012 [21]
Great -Horned Owl* (Bubo virginianus)*					9.9	±	2.2	6.0–14.0 (15)	Labella et al. 2012 [21]
Barn owl* (Tyto alba)*	3.6	±	2.2 (29)					Tonopen® XL	Beckwith et al. 2015 [49]
Scops owl *(Otus scops)*	1.0	±	0.5 (23)						Beckwith et al. 2015 [49]
Long -eared owl *(Asio Otus)*	1.25	±	1.00 (4)						Beckwith et al. 2015 [49]
Little owl *(Athena noctua)*	2.5	±	0.7 (4)						Beckwith et al. 2015 [49]
Eurasian eagle-owl *(Bubo bubo interpositus)*	12.0	±	7.0 (4)						Beckwith et al. 2015 [49]
Pharaoh eagle owl *(Bubo bubo ascalaphus)*	15	±	0 (2)						Beckwith et al. 2015 [49]
Black kite *(Milvus migrans)*	7.4	±	5.7 (10)						Beckwith et al. 2015 [49]
European Honey buzzard *(Pernis apivorus)*	7.5	±	2.2 (18)						Beckwith et al. 2015 [49]
Western march harrier *(Circus aeruginosus)*	12.0	±	5.6 (4)						Beckwith et al. 2015 [49]
Short-toed Snake-eagle *(Circaetus gallicus)*	7.5	±	3.5 (4)						Beckwith et al. 2015 [49]
Montagus Harrier *(Circus pygargus)*	8.0	±	2.8 (4)						Beckwith et al. 2015 [49]
Common buzzard *(Buteo buteo)*	13.7	±	4.4 (20)						Beckwith et al. 2015 [49]
Steppe buzzard *(Buteo buteo vulpinus)*	3.0	±	0 (2)						Beckwith et al. 2015 [49]
Long-legged Buzzard *(Buteo rufinus)*	12.5	±	10.0 (4)						Beckwith et al. 2015 [49]
Common kestrel *(Falco tinnunculus)*	5.8	±	4.0 (2)						Beckwith et al. 2015 [49]
Barbary falcon *(Falco pelegrinoides)*	3.0	±	0.0 (2)						Beckwith et al. 2015 [49]
*Lesser kestrel* *(Falco naumanni)*	2.0	±	0.0 (2)						Beckwith et al. 2015 [49]
Eastern Screech owl *(*Megascops asio)	2	<	median	2–6	9.0	±	1.8	(6–14) (22) TonoVet®-P	Harris et al. 2008 [2]
					14.0	±	2.4	(9–20) (22) TonoVet®‐D	Harris et al. 2008 [2]
American white pelicans (*Pelecanus erythrorhynchos*)					9.0	±	1.41	TonoVet®-P	Kinney et al. 2017 [25]
Great Rhea *(Rhea americana*)				21 OD 20 OS				Tonopen® XL	Church et al. 2013 [32]
Chicken *Gallus gallus domesticus*					17.51	±	0.13	(210) TonoVet®-Icare	Prashar et al. 2007 [1]
Great grey owls (*Strix nebulosa*)	9.8	±	2.8	5.0- 16.0 (23)	9.6	±	2.6	4.0- 14.0 TonoVet®-P, Icare	Wills et al. 2016 [15]
Snowy owls (*Bubo scandiacus*)	9.8	±	2.4	6.0- 15.0 (19)	9.1	±	1.9	4.0- 12.0 TonoVet®-P, Icare	Wills et al. 2016 [15]

The scarcity of ophthalmic findings in penguins makes it challenging to interpret these animals' ocular examination findings and diagnostic test results. Thus, it is important to establish reference values for routinely used ocular parameters such as IOP and STT in penguins. Several studies have been performed to determine STT and IOP values in the Humboldt penguin, macaroni penguin [6, 23], southern rockhopper penguin [6], black-footed penguin (*Spheniscus demersus*), gentoo penguin, king penguin and chinstrap penguin [3, 24, 32] (Table 6). Only Sheldon et al. [16] have attempted to establish reference values for tear production and IOP values with the STT and rebound tonometry, respectively, in wild Humboldt penguins in their natural habitat. Compared to the values previously detected by [16] in Humboldt penguins living in their natural habitat (Table 6), the present study demonstrated higher values (38.9 ± 13.2 mmHg) falling within a more extensive range (16–69 mmHg), which were attributed to the harsh Antarctic weather conditions characterized by sudden changes.

IOP measurements by rebound tonometry have been previously performed in the Humboldt penguin (*Spheniscus humboldti*) [16], macaroni penguin [6], southern rockhopper penguin [6, 34], black-footed penguin [3, 24], gentoo penguin [46]), king penguin and chinstrap penguin [32]. The mean IOP values of healthy macaroni and southern rockhopper penguins were 42.0 ± 9.7 mmHg and 32.9 ± 6.2 mmHg, respectively. No statistically significant difference was detected in these two penguin species for gender or the left/right eye. In previous research on the use of tonometry in penguins, the mean IOP value calculated for a healthy eye was determined to be above 28 mmHg and, thus, was significantly higher than values previously reported in several other avian species (Tables 6 and 7) [3, 6, 24]. A relatively lower mean IOP value of 20.4 ± 4.1 mmHg was reported for the Humboldt penguin. Still, it should be noted that this value was obtained using the applanation tonometry technique, which is known to yield significantly lower IOP values in penguins and other birds compared to rebound tonometry [12, 50]. Therefore, comparing penguin IOP values obtained with the same tonometry technique is required [8]. Although scarce, IOP values obtained with rebound tonometry have been reported for some penguin species (Table 7). The present study's results agree with previous studies on using TonoVet in penguins. Compared to the IOP ranges previously reported for other avian species, the mean IOP values determined in *Pygoscelis* penguins in the present study were higher (Tables 2 and 4).

Studies available on the use of STTs in penguins are limited to the macaroni penguin (and rockhopper penguin [6, 12, 16]. Of these studies, only two [12, 16] have reported STT values. Swinger et al. reported an STT range of 1–12 mm/min and a mean STT value of 6.5 ± 2.9 mm/min for the Humboldt penguin [12]. Different results have been reported for animals rehabilitated in freshwater and marine environments. Accordingly, researchers have reported mean STT values of 4.8 mm/min and 8.5 mm/min for penguins in experimental marine and freshwater environments. Thus, the values have demonstrated a significant difference between the two habitats. The mean STT value of freshwater penguins was two-fold that of marine penguins, which was attributed to differences between the supraorbital glands of these species [12]. However, the specific geographical structure and abrupt changes in weather conditions of the Antarctic Peninsula caused different numbers of samples to be taken from various locations. Despite the balanced distribution of standard deviations, a difference of 1/4 of the maximum value of 12 was observed between the minimum and maximum means (Table 3). In avian species, the harderian gland, situated near the base of the nictitating membrane, is the primary source of tear fluid [51, 52]. Harris et al. [2] suggested that owls and penguins produced a smaller volume of aqueous tear owing to the smaller size or absence of lacrimal glands than other birds [53]. Similarly, Meekins et al. [14] reported that tear production varied greatly among birds of assorted sizes and phylogenetic classifications. STT-II values previously reported for macaroni penguins and rockhopper penguins kept at zoos were found to be like the STT-I values detected in *Pygoscelis* penguins in the present study (Table 6).

In their research aimed at establishing STT and IOP ranges for some raptors, Barsotti et al. [48] determined the presence of significant inter-species differences. Compared to values previously reported for other avian species, the mean IOP and STT-1 values determined in the present study are similar, higher, or lower (Table 7). The differences observed could be related to a marine adaptation serving as an advantage to penguins during underwater diving and foraging.

The number of ophthalmic measurements performed in the various locations varies due to the abrupt changes in the Antarctic weather. While the number of penguins sampled in the Ardley SPA (Ardley III) for STT-1 measurements was 24, IOP values were measured in 33 penguins. High winds that blew during the visits, which made it exceedingly difficult to place the filter papers in the conjunctival fornix without causing any harm to the penguins, prevented the completion of the measurements in some animals. The restraint of penguins was not prolonged, and the measurements were not repeated to avoid any animal stress. Heavy rain during the visits to Harmony Point caused the tonometer to display values outside the normal range. As the repetition of tonometer measurements would require the prolonged restraint of the animals, causing increased stress that would prevent the achievement of accurate results, IOP values were not measured. Due to adverse weather conditions, ophthalmic measurements could not be made at Doumer Island/Yelchoo Base.

In the present study, it took time for the animals to calm down after being captured and physically restrained for clinical tests and observations. Given the data collected from the animals under physical restraint and to prevent any error, the data was collected from only one eye in each animal. Apart from two people required to restrain the animals and perform tests on them, placing the STT test strips in the conjunctival fornix was another significant difficulty. For the correct placement of the strips, it was required to open the eyelid and, at the same time, apply the test. Furthermore, given the small size of penguin eyes, it should be noted that placing the strips in the conjunctival fornix without touching the cornea is almost impossible and eventually irritates the eye and causes artifactitious tear production.

## Conclusions

In conclusion, this study presents both TP and IOP values measured with an STT-1 Tonovet® rebound tonometer and values detected in clinically healthy *Pygoscelis* penguins. We consider these aspects important for researchers and practitioners when diagnosing subtle pathological changes in tear production. Moreover, there is a need for further veterinary research on ocular surface measurements in penguin species living in their natural habitat other than those investigated in the present study. Also, further research is required on varied species in colonies on different islands. This study will constitute a reference for future studies conducted in various locations and with varied species.

## Materials and methods

### Locations and times of study

A bilateral cooperation project titled "The cytological, microbiological and ophthalmic evaluation of ocular surface samples from Antarctic penguins," conducted with the Chilean Antarctic Institute (INACH) within the scope of the 55^th^ Antarctic Scientific Expedition (ECA55), was implemented in the 2018–2019 period, under the Third National Antarctic Scientific Expedition (TAE III) organized by the Polar Research Centre (PolRec) of Istanbul Technical University. The project was approved under the Turkish Antarctic Programme (Antarctic Specially Protected Area, ASPA, No: 150, Permit N̊- 21- 2019,' Permit N̊- 07- 2019). The locations visited (between 62° 06' S—058° 09' W and 64° 52' S—063° 32' W) in the study included Harmony Point/Nelson Island (Antarctic Specially Protected Area No. 133) (ASPA 133), Doumer Island/Yelchoo Base, Cabo Legoupil/General Bernardo O'Higgins Base, Ardley Island (Antarctic Specially Protected Area No. 150) (ASPA 150) and Lions Rump, King George Island (Antarctic Specially Protected Area No. 151) (ASPA 151, Table 1).

**Fig. 1:**
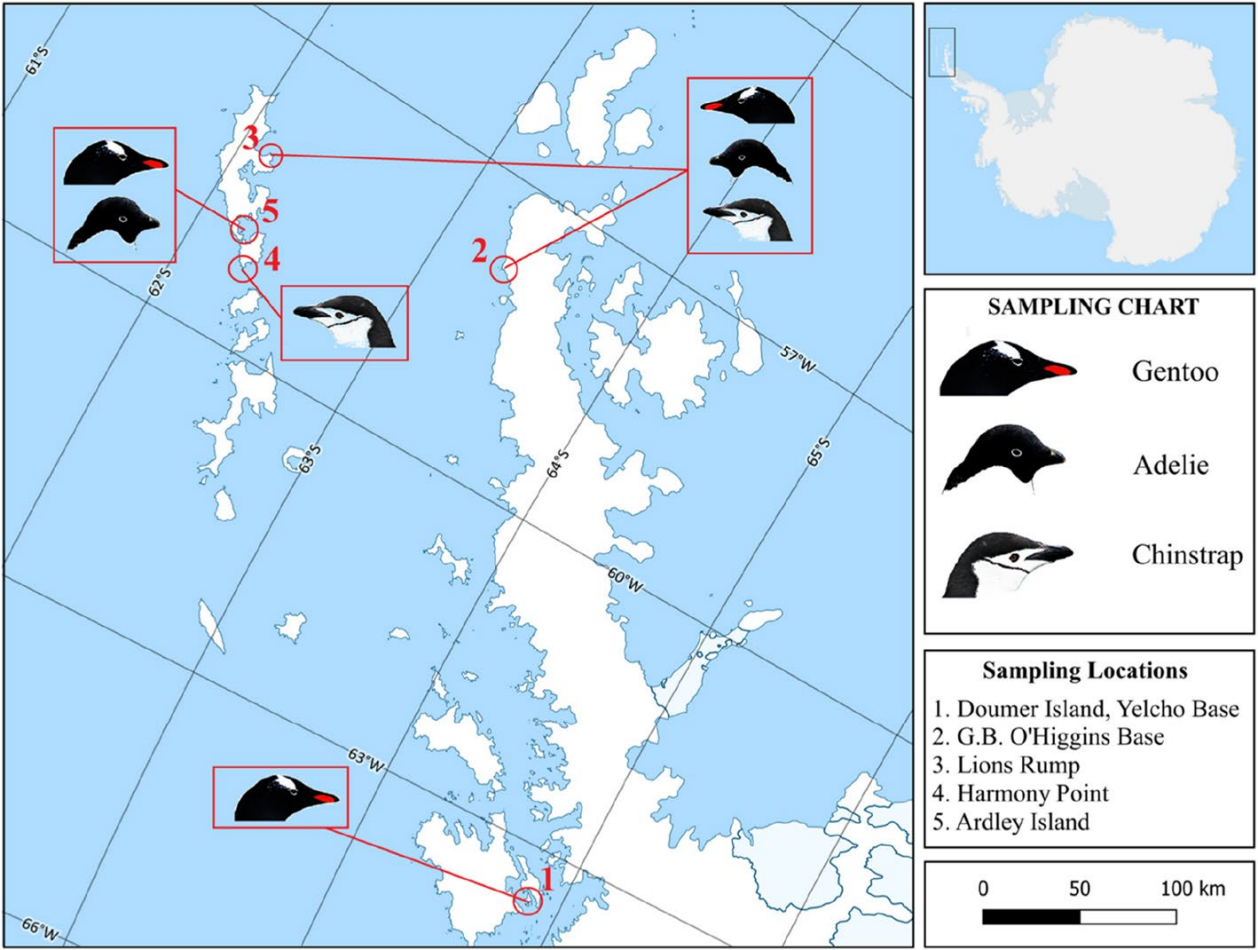
Location of the study area. Red dots indicate the location of the colonies of *Pygoscelis* penguins

The islands were visited three times for sampling. A zodiac was used for transport from the ship to the locations. A different entry point was used during each visit. According to the penguin species to be sampled, either the Louis entry point (Refuge Balive, Brazilian Refuge "Astronomo Cruls" -R1ANF/P, Ardley I), Braillard entry point (Julio Ripomonti Refuge, Ardley II), or Faro entry point (Ardley III) were used (Fig. 1).

### Method of capture and handling

The capture and handling of the penguins were performed as described by González-Acuña et al. [54] and following the standard methods laid down for the Ecosystem Monitoring Programme by the Commission for the Conservation of the Antarctic Marine Life Resources [55]. During the sampling procedure, the penguins were restrained in an upright vertical position by applying gentle pressure to the wings, base of the skull, and beak. During the ophthalmic examination, the penguins were restrained manually in a facedown position. According to the standard procedure, the captor restrained each penguin in such a way that the ventrum of the animal lay on the legs. While holding the animal's wings with one hand, the captor fixed the legs in an extended position with his other hand. In the meantime, the penguin's body leaned onto the captor's abdomen. The same researcher performed all diagnostic tests to avoid measurement failure or technical error [6]. For data collection, only one ocular sample was taken from each physically restrained animal in the shortest time possible. Penguins away from their nesting sites, going to feed or returning from the ocean, were selected for sampling. Penguins were caught with a tool with a long handle and a wide net (such as a fish or butterfly net). The wings were held with one arm and the feet with the other arm of the researcher without causing much irritation. Then, they were prepared for sampling by holding the feet and wings in a horizontal shape. Each penguin was captured manually and restrained for approximately 6 min. After collecting samples and taking measurements, birds were immediately released [47].

### Ophthalmic tests

**Fig. 2:**
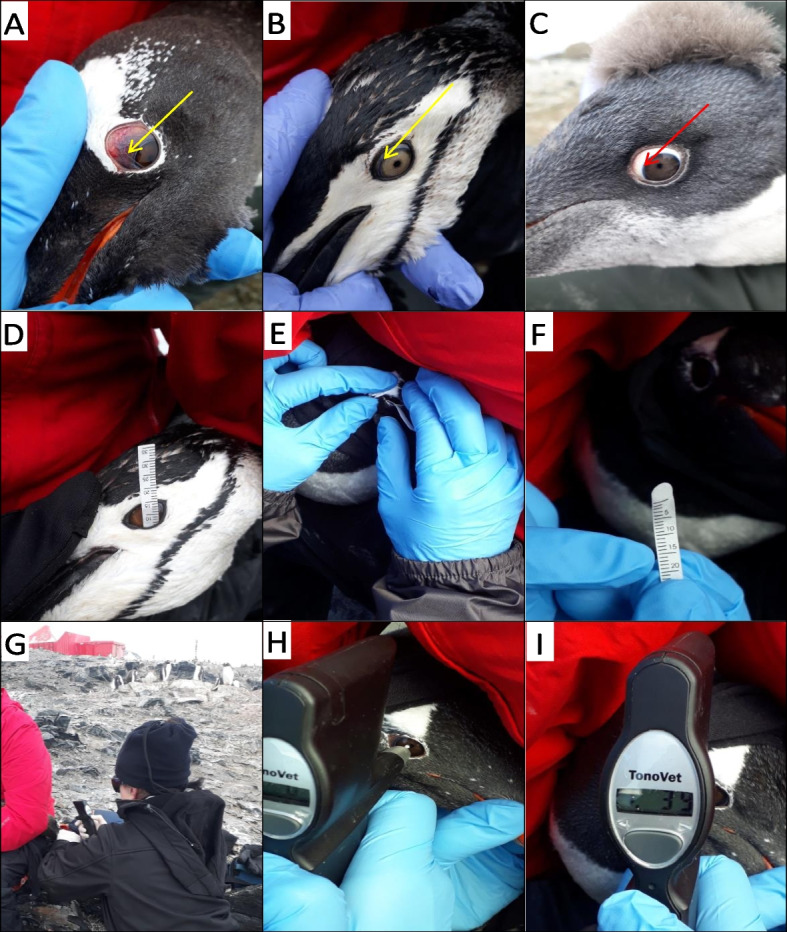
Normal eye of three species of *Pygoscelis* penguins. Nictitating membranes can be seen slightly covering the eye of **A**) Gentoo penguin, *Pygoscelis papua* (yellow arrow), **B**) Chinstrap penguin, *Pygoscelis antarctica* (yellow arrow), **C**) Adélie penguin, *Pygoscelis adeliae* (red arrow)*.* The transparent nictitating membrane is apparent ventromedially (arrows). **D**-**I** Ophthalmic evaluation of *Pygoscelis* penguins that was subjected to STT-1 (**D**-**F**), with the TonoVet® (Icare®, Finland, Oy) rebound tonometer (**G**-**I**). **D** Schirmer's tear test being performed on an Adélie penguin, *Pygoscelis adeliae.* The strip had been inserted into the inferior lateral conjunctival fornix. Strips were best situated in the temporal third of the ventral conjunctival fornix to avoid displacement by the nictitans. **E** The image depicts the placement of a Schirmer's tear strip in the lower conjunctival fornix on a gentoo penguin **F**) The strip was then removed, and the tear production was measured in millimeters **H**-**I**) Manual restraint of penguins during the exam. Measurement of intraocular pressure using a veterinary rebound tonometer on the P setting in a *Pygoscelis* penguin. The Icare® rebound tonometer measuring intraocular pressure in the left eye of a gentoo penguin. The probe is positioned approximately 5 mm from the corneal surface before deployment

Owing to the specific geographical structure of the Antarctic Peninsula and the sudden changes in its weather conditions, the penguins could not be physically restrained and subjected to ophthalmic measurements in a closed, protected, and quiet environment. The penguins were macroscopically examined for signs of possible ocular infection and sight impairment. For this purpose, the menace reflex test was conducted bilaterally by waving a hand in front of both eyes. To avoid any air flow-related false positive result, the hand was waved at a distance of at least 30 mm to the tested eye. The menace reflex was considered present when the penguin responded to the visual threat by continuous head movement, blinking of the eye, or opening its mouth in a threatening manner. The reflex was considered inconsistent when the penguin moved its head or blinked its eye once and did not repeat this response to continuous hand waving [29]. In all the penguin species examined, the third eyelid was transparent and displayed its typical structure (Fig. 2A-C).

The animals were exposed to minimal stress under physical restraint. Yet, a significant difficulty in applying the standard procedure was not allowing the time required for the penguins to calm down. It also negatively affected the collection of accurate data on their blinking frequency. Thus, the ocular tests were applied to the left eye only. Given these requirements and to minimize the handling period, tear production measurements with the STT-I (129 penguins) and IOP measurements (120 penguins) with the TonoVet® tonometer were performed in only the left eye of each penguin.

### Method of STT

A single person performed the STT-I, according to the manufacturer's instructions, using strips of sterile standardized filter paper, 35 mm long and 5 mm wide. The standardized strips (Schirmer's-Tränentest®; Vet Eickemeyer, Tuttlingen, Germany) were placed in the lower conjunctival fornix for one minute. The strips were bent at the dented part and, employing dry forceps, the bent part was placed in the exterior one-third of the lateral canthus of the left lower eyelid (Fig. 2D-F). The amount of tear absorbed by the strip was measured in millimeter/minute with the aid of the millimetric scale on the strip. Care was taken to handle the strips only by the sides to avoid contact with any object or moisture before sampling [16, 46].

### Method of intraocular pressure

During the macroscopic examination, the ophthalmic measurements, and tests, the penguins were manually restrained in a facedown position by the captor. The penguin's beak was held with one hand, and gentle pressure was applied to the occipital base of the skull. The IOP readings were performed with a rebound tonometer (TonoVet®; Icare, Helsinki, Oy, Espoo, Finland), using a P calibration setting installed in the tonometer by the manufacturer. Measurements were performed according to the manufacturer's instructions, maintaining the tonometer in a horizontal position, and holding the probe at 4–8 mm from the cornea. Measurements were made from the cornea censer of the left eye. Care was taken to ensure that the probe came in contact with the cornea only when the third eyelid (nictitating membrane) (Fig. 2A-C) had been contracted. To avoid readings of the third eyelid and the generation of false results while collecting data. The tonometer digitally displayed the IOP value on its screen each time the cornea was touched. After the fifth touch, an average of the previous five readings was generated automatically. The average measurement was calculated by the tonometer (highest and lowest values excluded) [16]. Readings could not be performed in some animals, either due to their excessive movement under restraint or due to stormy, rainy, or snowy weather. Therefore, only data about the animals in which the five readings could be completed were analyzed (Fig. 2G-I). All the penguins recruited in the study were free of any ocular lesions.

### Statistical analyses

The study data were collected from 04 January to 06 February 2019. Statistical analyses were conducted using the TURCOSA cloud (Turcosa Analytics Ltd. Co., Turkey) statistical software (https://turcosa.com.tr/). The normal distribution of numerical variables was analyzed with the Shapiro–Wilk test of normality and Q-Q graphics. One-way analysis of variance (ANOVA) was used for the comparison of more than two groups (Comparison of STT (mm/min) and IOP (mmHg) by locations and species). Student's t-test was used to compare two independent groups (Comparison of IOP amounts (mmHg) by species). Tukey's test was used as a multiple comparison test. For any comparison, if the *p*-value obtained was lower than the significance level used (*α* < 0.05), it was concluded with 95% confidence that there were statistically significant differences between the values compared.

## Data Availability

The data sets generated and/or analyzed during the current study are available from the corresponding author upon reasonable request.

## Abbreviations

TP: Tear production
IOP: Intraocular pressure
IUCN: The International Union for Conservation of Nature
STT-1: Schirmer’s tear test
*P*: *Pygoscelis*
SPA: Specially Protected area
INACH: Chilean Antarctic Institute
ASPA: Antarctic Specially Protected Area
TAE: Turkish Antarctic Expedition
PolRec: Polar Research Centre
